# Increased Income for Charitable Institutions

**Published:** 1916-08-26

**Authors:** 


### INCREASED INCOME FOR CHARITABLE INSTITUTIONS.

### The Fulfilment of a Patriotic Duty.

(FROM A CORRESPONDENT.)

The latest extension of the Treasury scheme for the mobilisation of securities, combined as it is with the fulfilment of a patriotic duty, deserves the most careful consideration on the part of hospital trustees, to whom it should appeal as a means of providing increased income.

The scheme which is now submitted by the Treasury is part of the Government plan for the regulation of foreign exchanges, and is to be known as Scheme B. Shortly the essential features are:—

(*a*) Holders of American Dollar Securities deposited under Scheme A for two years may not later than September 14 transfer their deposits to Scheme B.

(*b*) Deposits of new securities under Scheme B are transferable to the Treasury for a period of about $5\frac{1}{2}$ years (*i.e.*, five years from March 31, 1917), the Treasury having the right to return the securities to the original holders on or after March 31, 1919, on three months' notice. In the event of any of the securities being paid off during the period of the deposit the amount received will be paid to the depositor.

(*c*) While securities are held on deposit the lender will receive his interest and dividends, and in consideration for the loan a payment of $\frac{1}{2}$ per cent. per annum on the face value of the securities.

(*d*) At the end of the period of deposit the securities or other securities of the same description and of the same nominal amount will be returned to the depositor.

(*e*) The Treasury will have the right to dispose of the securities should they find it neccessary to do so, in which case the lender will continue to receive the dividends he would ordinarily have enjoyed, and at the end of the period of the loan the Treasury will return the securities or other securities of equal value, and to the same nominal amount, or, at the holder's option, the deposit value of the securities, *plus* 5 per cent. on the value, and accrued interest from the preceding interest date—*i.e.*, from the date of the last-paid dividend.

(*f*) In the case of securities repayable at a fixed date or by drawings, the Treasury will, when making payment to the depositor, if the deposit value, *plus* 5 per cent. (clause *e*), is less than the redemption value (the amount received on repayment or drawing), add a further amount to equal the enhanced value on redemption.

(*g*) Deposit value means the quoted price on the London Stock Exchange (less accrued interest where this is included in the quoted prices), as on the day preceding the present notice—*i.e.*, August 11, 1912.

(*h*) Holders of bearer bonds will lodge their bonds and coupons with the Treasury, and holders of registered stock or bonds will be required to execute at the Government expense a transfer of the securities to the Treasury, upon which the holder's name will be inscribed in a special register, and a certificate of entry will then be issued.

(*i*) The certificates thus issued will be saleable on the Stock Exchange.

(*j*) Interest and dividends on the deposited securities (*plus* $\frac{1}{2}$ per cent. per annum on the face value) will be payable by warrant by the Treasury as soon as received.

(*k*) Trustees are enabled to take advantage of the scheme without liability for any loss which may arise.

The terms now offered by the Treasury should appeal strongly to trustees, offering as they do an increased interest of $\frac{1}{2}$ per cent. per annum on the face value of the securities deposited (that is the nominal value of the holding, not the purchase price), and, if a sale is effected, an enhancement of 5 per cent. on the value as on August 11.

It is not often that "new lamps for old" are obtainable for the asking, and it would appear that the opportunity to secure an increased income, and at the same time fulfil a patriotic duty, should be largely taken advantage of by hospital trustees.

The list of securities scheduled under the scheme covers a wide field, and includes high-yielding securities hitherto much sought for by trustees, such as Argentine Government Loans, Dominion of Canada 3, $3\frac{1}{2}$, $3\frac{3}{4}$, 4, and $4\frac{1}{2}$ per cent. stocks and bonds, Canadian railway debentures, and other loans.

A careful persual of the list and of the terms of deposit is commended to hospital trustees, especially as a strong objection to the Treasury right to dispose of securities has been met by the Treasury amplifying clause (*e*) by undertaking to hand over any profit actually arising from the sale of deposited securities.

The columns of The Hospital are open to the discussion of any points which may present themselves to hospital trustees and administrators on this important matter, for it cannot be doubted that the total values of Schedule B securities held by hospital trustees are very considerable, and would be of great help to the nation in the Treasury plan to regulate foreign exchanges.

